# Supplementary material for: Identification and Prioritization of Environmental Reproductive Hazards: A First Step in Establishing Environmental Perinatal Care
Source: Int J Environ Res Public Health. 2019 Jan 28;16(3):366. doi: 10.3390/ijerph16030366 (PMC6388153; doi:10.3390/ijerph16030366)
Supplement: Supplementary file 1 [file ijerph-16-00366-s001.pdf]

Supplemental Table S1. Full list of environmental chemical hazards on reproduction.

Category	CAS Number	Name of the reproductive risk factor
1	630-08-0	carbon monoxide
1	25808-74-6	lead hexafluorosilicate
1	94551-87-8	slimes and sludges, copper electrolyte refining, decopperised
1	68130-19-8	silicic acid, lead nickel salt
1		lead compounds with the exception of those specified elsewhere in the CLP regulation
1		lead alkyls
1	13424-46-9	lead diazide, lead azide
1	7758-97-6	lead chromate
1	301-04-2, 6080-56-4	lead di(acetate)
1	7446-27-7	trilead bis(orthophosphate)
1	1335-32-6	lead acetate, basic
1	17570-76-2	lead(II) methanesulphonate
1	1344-37-2	lead sulfochromate yellow
1	12656-85-8	lead chromate molybdate sulfate red
1	7784-40-9	lead hydrogen arsenate
1	7439-92-1	lead
1	96-12-8	1,2-dibromo-3-chloropropane
1	102-06-7	1,3-diphenylguanidine
1	629-14-1	1,2-diethoxyethane
1		abacavir
1	68049-83-2	azafenidin
1	15245-44-0	lead styphnate
1	13517-20-9, 15120-21-5	perboric acid (H3BO2(O2)), monosodium salt, trihydrate
1	10332-33-9, 11138-47-9, 12040-72-1, 37244-98-7	perboric acid, sodium salt, monohydrate, perboric acid, sodium salt
1	12008-41-2, 12280-03-4	disodium octaborate tetrahydrate, disodium octaborate anhydrous
1	17804-35-2	benomyl
1	485-31-4	binapacryl
1	56073-10-0	brodifacoum
1	27366-72-9	(dimethylamino)thioacetamide hydrochloride
1	28772-56-7	bromadiolone
1	84245-12-5	[6,9-dihydro-9-[[2-hydroxy-1-(hydroxymethyl)ethoxy]methyl]-6-oxo-1H-purin-2-yl]acetamide
1	74-83-9	bromomethane
1	75113-37-0	dibutyltin hydrogen borate
1	10043-35-3	boric acid
1	1303-86-2	diboron trioxide

1	12267-73-1	tetraboron disodium heptaoxide, hydrate
1	13840-56-7	orthoboric acid, sodium salt
1	1303-96-4, 1330-43-4, 12179-04-3	disodium tetraborate decahydrate
1	7632-04-4, 10332-33-9, 10486-00-7	sodium peroxometaborate
1	106-94-5	1-bromopropane
1	7778-50-9	potassium dichromate
1	7789-09-5	ammonium dichromate
1	7789-12-0, 10588-01-9	sodium dichromate
1	7775-11-3	sodium chromate
1	7646-79-9, 7791-13-1	cobalt dichloride
1	10026-24-1, 10124-43-3	cobalt sulfate
1	71-48-7, 6147-53-1	cobalt di(acetate)
1	10026-22-9, 10141-05-6	cobalt dinitrate
1	513-79-1	cobalt carbonate
1	13463-39-3	tetracarbonylnickel
1	12054-48-7	nickel dihydroxide
1	11113-74-9	nickel hydroxide
1	7786-81-4, 10101-97-0, 10101-98-1	nickel sulfate
1	12607-70-4	[carbonato(2-)]tetrahydroxytrinickel
1	65405-96-1	[μ-[carbonato(2-)-O:O′]] dihydroxy trinickel
1	16337-84-1	carbonic acid, nickel salt
1	3333-67-3	nickel carbonate
1	7718-54-9, 7791-20-0	nickel dichloride
1	14216-75-2	nitric acid, nickel salt
1	13138-45-9, 13478-00-7	nickel dinitrate
1	92129-57-2	slimes and sludges, copper electrolytic refining, decopperised, nickel sulfate
1	13637-71-3	nickel diperchlorate
1	15699-18-0	diammonium nickel bis(sulfate)
1	13842-46-1	nickel dipotassium bis(sulfate)
1	13770-89-3	nickel bis(sulfamidate)
1	14708-14-6	nickel bis(tetrafluoroborate)
1	68134-59-8	formic acid, copper nickel salt
1	15843-02-4	formic acid, nickel salt
1	3349-06-2	nickel diformate
1	14998-37-9	nickel acetate
1	373-02-4, 6018-89-9	nickel di(acetate)
1	553-71-9	nickel dibenzoate
1	3906-55-6	nickel bis(4-cyclohexylbutyrate)
1	2223-95-2	nickel(II) stearate
1	16039-61-5	nickel dilactate

1	4995-91-9	nickel(II) octanoate
1	13462-88-9	nickel dibromide
1	13462-90-3	nickel diiodide
1	10028-18-9	nickel difluoride
1	11132-10-8	nickel potassium fluoride
1	26043-11-8	nickel hexafluorosilicate
1	15060-62-5	nickel selenate
1	13689-92-4	nickel dithiocyanate
1	15586-38-6	nickel dichromate
1	67952-43-6	nickel dichlorate
1	71720-48-4	ethyl hydrogen sulfate, nickel(II) salt
1	14550-87-9	nickel dibromate
1	93983-68-7	dimethylhexanoic acid nickel salt
1	84852-36-8	(isodecanoato-O)(isononanoato-O)nickel
1	84852-35-7	(isooctanoato-O)(neodecanoato-O)nickel
1	84852-37-9	nickel bis(isononanoate)
1	84852-39-1	(2-ethylhexanoato-O)(isodecanoato-O)nickel
1	13654-40-5	nickel(II) palmitate
1	52625-25-9	nickel 3,5-bis(tert-butyl)-4-hydroxybenzoate (1:2)
1	93920-10-6	nickel(II) neononanoate
1	71957-07-8	bis(D-gluconato-O1,O2)nickel
1	84776-45-4	fatty acids, C8-18 and C18-unsatd., nickel salts
1	93920-09-3	nickel(II) neoundecanoate
1	3349-08-4	nickel(II) propionate
1	7580-31-6	2-ethylhexanoic acid, nickel salt
1	39819-65-3	nickel bis(benzenesulfonate)
1	18283-82-4	citric acid, ammonium nickel salt
1	85508-44-7	nickel(II) neodecanoate
1	85508-43-6	nickel(II) isodecanoate
1	85551-28-6	(isononanoato-O)(neodecanoato-O)nickel
1	29317-63-3	nickel(II) isooctanoate
1	85508-46-9	(isononanoato-O)(isooctanoato-O)nickel
1	85508-45-8	(2-ethylhexanoato-O)(isononanoato-O)nickel
1	4454-16-4	nickel bis(2-ethylhexanoate)
1	85166-19-4	(isodecanoato-O)(isooctanoato-O)nickel
1	91697-41-5	fatty acids, C6-19-branched, nickel salts
1	16083-14-0	nickel(II) trifluoroacetate
1	22605-92-1	citric acid, nickel salt
1	85135-77-9	(2-ethylhexanoato-O)(neodecanoato-O)nickel
1	51818-56-5	neodecanoic acid, nickel salt
1	18721-51-2	nickel(II) hydrogen citrate
1	27637-46-3	nickel isooctanoate
1	72319-19-8	2,7-naphthalenedisulfonic acid, nickel(II) salt

1	1303-00-0	gallium arsenide
1	7790-79-6	cadmium fluoride
1	10108-64-2, 35658-65-2	cadmium chloride
1	7790-84-3, 10124-36-4, 31119-53-6	cadmium sulphate
1		tributyltin compounds, with the exception of those specified elsewhere in this annex
1	683-18-1	dibutyltin dichloride
1	15571-58-1	2-ethylhexyl 10-ethyl-4,4-dioctyl-7-oxo-8-oxa-3,5-dithia-4-stannatetradecanoate
1	77-58-7	dibutyltin dilaurate
1	7439-97-6	Mercury and mercury compounds
1	50-32-8, 63466-71-7	benzo[a]pyrene
1	111-41-1	2-(2-aminoethylamino)ethanol
1	96-18-4	1,2,3-trichloropropane
1	106-91-2	2,3-epoxypropyl methacrylate
1	109-86-4, 109-87-5	2-methoxyethanol
1	110-80-5	2-ethoxyethanol
1	110-71-4	1,2-dimethoxyethane
1	556-52-5	2,3-epoxypropan-1-ol
1	78-93-3	2-butanone
1	1589-47-5	2-methoxypropanol
1	111-96-6	bis(2-methoxyethyl) ether
1	75-26-3	2-bromopropane
1	112-49-2	1,2-bis(2-methoxyethoxy)ethane
1	71868-10-5	2-methyl-1-(4-methylthiophenyl)-2-morpholinopropan-1-one
1	6807-17-6	4,4-isobutylethylidenediphenol
1	80-05-7	bisphenol A
1		ado-trastuzumab emtansine
1	79-06-1, 122775-19-3	acrylamide
1	75-15-0	carbon disulphide
1	67-66-3	chloroform
1	32536-52-0	diphenylether; octabromo derivate
1	96-45-7	ethylene thiourea
1	10605-21-7	carbendazim
1	127-19-5	dimethylacetamide
1	624-83-9	methyl isocyanate
1	68515-50-4	1,2-benzenedicarboxylic acid, dihexyl ester, branched and linear
1	151798-26-4	2-[2-hydroxy-3-(2-chlorophenyl)carbamoyl-1-naphthylazo]-7-[2-hydroxy-3-(3-methylphenyl)carbamoyl-1-naphthylazo]fluoren-9-one
1	94723-86-1	2-butyryl-3-hydroxy-5-thiocyclohexan-3-yl-cyclohex-2-en-1-one

1	110-49-6	2-methoxyethyl acetate
1	111-15-9	2-ethoxyethyl acetate
1	16118-49-3	carbetamide
1	2687-91-4	N-ethyl-2-pyrrolidone
1	3691-35-8	chlorophacinone
1	80387-97-9	2-ethylhexyl[[[3,5-bis(1,1-dimethylethyl)-4-hydroxyphenyl]methyl]thio]acetate
1	68515-42-4	1,2-benzenedicarboxylic acid
1	70657-70-4	2-methoxypropyl acetate
1	5836-29-3	coumatetralyl
1	66-81-9	cycloheximide
1	625-45-6	methoxyacetic acid
1	84777-06-0	1,2-benzenedicarboxylic acid, dipentylester, branched and linear
1	85-68-7	benzyl butyl phtalate
1	94361-06-5	cyproconazole
1	56073-07-5	difenacoum
1	117-81-7	bis(2-ethylhexyl) phthalate
1	117-82-8	bis(2-methoxyethyl) phthalate
1	84-74-2, 93952-11-5	dibutyl phthalate
1	84-61-7	dicyclohexyl phthalate
1	84-75-3	dihexyl phthalate
1	84-69-5	diisobutyl phthalate
1	7425-14-1	2-ethylhexyl-2-ethylhexanoate
1	605-50-5	diisopentylphthalate
1	3108-42-7	ammonium nonadecafluorodecanoate
1	29081-56-9	ammonium perfluorooctane sulfonate
1	3825-26-1	ammoniumpentadeca- fluorooctanoate
1	70225-14-8	diethanolamine perfluorooctane sulfonate
1	29457-72-5	lithium perfluorooctane sulfonate
1	143860-04-2	3-ethyl-2-methyl-2-(3-methylbutyl)-1,3-oxazolidine
1	84-74-2	Di-n-butyl phthalate (DBP)
1	335-76-2	nonadecafluorodecanoic acid
1	375-95-1	perfluorononan-1-oic acid
1	98-73-7	4-tert-butylbenzoic acid
1	104653-34-1	difethialone
1	39300-45-3	dinocap
1	4149-60-4	perfluorononan-1-oic acid ammonium salts
1	21049-39-8	perfluorononan-1-oic acid sodium salts
1	1763-23-1	perfluorooctane sulfonic acid
1	131-18-0	di-n-pentyl phthalate
1	335-67-1	perfluorooctanoic acid
1	2795-39-3	potassium perfluorooctanesulfonate
1	3830-45-3	sodium nonadecafluorodecanoate

1	98-95-3	nitrobenzene
1	88-85-7	dinoseb
1	1420-07-1	dinoterb
1	106325-08-0, 133855-98-8	epoxiconazole
1	37894-46-5	etacelasil
1	90035-08-8	flocoumafen
1	69806-50-4, 79241-46-6	fluazifop-butyl
1	103361-09-7	flumioxazin
1	199327-61-2	7-methoxy-6-(3-morpholin-4-yl-propoxy)-3H-quinazolin-4-one
1	3724-43-4	chloro-N,N-dimethylformiminium chloride
1	85509-19-9	flusilazole
1	5571-36-8	cyclic 3-(1,2-ethanediylacetale)-estra-5(10),9(11)-diene-3,17-dione
1	592-62-1	methyl-ONN-azoxymethyl acetate
1	77182-82-2	glufosinate ammonium
1	288-32-4	imidazole
1	74499-35-7	phenol, (tetrapropenyl) derivatives
1	65277-42-1	ketoconazole
1	330-55-2	linuron
1	1836-75-5	nitrofen
1	119738-06-6	quizalofop-p-téfuryl
1		salts and esters of dinoseb, with the exception of those specified elsewhere in this Annex
1	65996-93-2	pitch, coal tar, high-temp.
1	183196-57-8	potassium 1-methyl-3-morpholinocarbonyl-4-[3-(1-methyl-3-morpholinocarbonyl-5-oxo-2-pyrazolin-4-ylidene)-1-propenyl]pyrazole-5-olate
1		salts and esters of dinoterb
1		reaction mass of: 1,3,5-tris(3-aminomethylphenyl)-1,3,5-(1H,3H,5H)-triazine-2,4,6-trione
1	105024-66-6	silafluofen
1	111988-49-9	thiacloprid
1	148-24-3	quinolin-8-ol
1	55219-65-3	triadimenol
1	68-12-2	dimethylformamide
1	107-21-1	Ethylene glycol (ingested)
1	75-12-7	formamide
1	591-78-6	hexan-2-one
1	67-56-1	methanol
1		reaction mass of: 4-[[bis-(4-fluorophenyl)methylsilyl]methyl]-4H-1,2,4-triazole
1		reaction mass of: disodium 4-(3-ethoxycarbonyl-4-(5-(3-ethoxycarbonyl-5-hydroxy-1-(4-sulfonatophenyl)pyrazol-4-yl)penta-2,4-dienylidene)-4,5-dihydro-5-oxopyrazol-1-yl)benzenesulfonate

1		phenol, 2-dodecyl-, branched
1	24602-86-6	tridemorph
1	68694-11-1	triflumizole
1		phenol, 3-dodecyl-, branched
1	210555-94-5	phenol, 4-dodecyl-, branched
1	79-16-3	methylacetamide
1	61571-06-0	tetrahydrothiopyran-3-carboxaldehyde
1	121158-58-5	phenol, dodecyl-, branched
1	106-50-3	p-phenylenediamine
1	57044-25-4	R-glycidol
1	62-56-6	thiourea
1	154229-18-2	Abiraterone acetate
1	115-96-8	tris(2-chloroethyl)phosphate
1	60-00-4	EDTA
1	50471-44-8	vinclozolin
1	123-39-7	methylformamide
1	25155-23-1	trixylyl phosphate
1	872-50-4	N-Methylpyrrolidone
1	1330-43-4	di-sodium tetraborate anhydrous
1		acitretin
1	10043-35-3 ; 11113-50-1	boric acid
1	15120-21-5	sodium perborate
1	97-99-4	tetrahydro-2-furylmethanol
1	645-05-6	altretamine
1		afatinib
1		anastrozole
1	1327-53-3	arsenic trioxide
1		axitinib
1		azacitidine
1		belinostat
1		bendamustine
1		bexarotene
1		bicalutimide
1		bleomycin
1		bortezomib
1		bosutinib
1		brentuximab vedotin
1		busulfan
1		cabazitaxel
1		cabozantinib
1		capecitabine
1	41575-94-4	Carboplatin
1		carfilzomib

1		carmustine
1	305-03-3	Chlorambucil
1		cisplatin
1	4291-63-8	Cladribine
1		clofarabine
1		crizotinib
1	147-94-4	Cytarabine
1		dabrafenib
1	4342-03-4	Dacarbazine
1		dactinomycin
1		dasatinib
1		decitabine
1		degarelix
1		docetaxel
1	82413-20-5	droloxifen
1		enzalutamide
1		epirubicin
1		eribulin
1		erlotinib
1		estramustine
1	33419-42-0	Etoposide
1		everolimus
1		exemestane
1		floxuridine
1		fludarabine
1	51-21-8	Fluorouracil
1	13311-84-7	Flutamide
1		fulvestrant
1		gemcitabine
1		gemtuzumab ozogamicin
1	127-07-1	Hydroxyurea
1		idarubicin
1	3778-73-2	Ifosfamide
1		imatinib
1		irinotecan
1		ixabepilone
1		letrozole
1		lomustine
1		mechlorethamine
1	148-82-3	Melphalan
1	6112-76-1	Mercaptopurine
1	59-05-2	Methotrexate
1		mitomycin

1		mitotane
1		nelarabine
1		nilotinib
1		omacetaxin
1		oxaliplatin
1	33069-62-4	Paclitaxel
1		pazopanib
1		pemetrexed
1	53910-25-1	Pentostatin
1	380610-27-5	Pertuzumab
1		pomalidomide
1		ponatinib
1		alitretinoin
1		ambrisentan
1		apomorphine
1	446-86-6	Azathioprine
1		bosentan
1	298-46-4	Carbamazepine
1		cetrorelix
1		chloramphenicol
1		choriogonadotropin
1	113852-37-2	Cidofovir
1	50-41-9	clomiphene
1	57109-90-7	Clorazepate dipotassium
1	64-86-8	colchicine
1	50-18-0	Cyclophosphamide (anhydrous)
1		cyclosporine
1	23541-50-6	Daunorubicin hydrochloride
1		deferiprone
1		dexrazoxane
1	56-53-1	Diethylstilbestrol (DES)
1		dinoprostone
1		divalproex
1	25316-40-9	Doxorubicin hydrochloride (Adriamycin)
1		dronedarone
1		dutasteride
1		entecavir
1		ergonovine/methylergonovine
1		eslicarbazepine
1		estradiol
1		estrogen/ progesterone combinations ; estrogens, esterified ; estrogens, conjugated
1	7280-37-7	Estropipate

1		finasteride
1		fingolimod
1		fluconazole
1	76-43-7	Fluoxymesterone
1		fosphenytoin
1	82410-32-0	Ganciclovir
1		ganirelix
1		gonadotropin, chorionic
1	65807-02-5	goserelin
1	220810-26-4	Histrelin acetate
1		icatibant
1		leflunomide
1		lenalidomide
1	74381-53-6	Leuprolide acetate
1		liraglutide
1		lomitapide
1		macitentan
1	71-58-9	Medroxyprogesterone acetate
1	595-33-5	Megestrol acetate
1	9002-68-0	Menotropins
1	60-56-0	Methimazole
1	58-18-4	Methyltestosterone
1		mifepristone
1		mipomersen
1	59122-46-2	Misoprostol
1	70476-82-3	Mitoxantrone hydrochloride
1		mycophenolate mofetil
1		mycophenolic acid
1	86220-42-0	Nafarelin acetate
1	10024-97-2	nitrous oxide
1		ospemifene
1		oxcarbazepine
1		oxytocin
1		palifermin
1		paliperidone
1		pamidronate
1		paroxetine
1		pasireotide
1		peginesatide
1		pentetate calcium trisodium
1		phenoxybenzamine
1		n-pentyl-isopentylphthalate
1		phenytoin

1		pralatrexate
1	54-91-1	Pipobroman
1		plerixafor
1		regorafenib
1		romidepsin
1	366-70-1	Procarbazine hydrochloride
1		sorafenib
1		progestins
1		raloxifene
1		rasagiline
1		sunitinib
1		temozolomide
1	36791-04-5	Ribavirin
1		riociguat
1		risperidone
1		sirolimus
1	64-17-5	Ethanol
1	108-88-3	Toluene
1		temsirolimus
1	29767-20-2	Teniposide
1		spironolactone
1	154-42-7	Thioguanine
1	18883-66-4	Streptozocin (streptozotocin)
1		tacrolimus
1	54965-24-1	Tamoxifen citrate
1		topotecan
1		toremifene
1		telavancin
1	846-50-4	Temazepam
1		teriflunomide
1		trametinib
1		testosterone
1		triptorelin
1	50-35-1	Thalidomide
1		valrubicin
1		tofacitinib
1	97240-79-4	Topiramate
1		tretinoin
1		ulipristal
1		vandetanib
1		vemurafenib
1		vinorelbine
1	66-75-1	Uracil mustard

1		valganciclovir
1	99-66-1	Valproate (valproic acid)
1		vigabatrin
1	143-67-9	Vinblastine sulfate
1	2068-78-2	Vincristine sulfate
1	879085-55-9	Vismodegib
1		voriconazole
1		vorinostat
1	1330-20-7	xylenes
1	81-81-2 ; 5543-57-7 ; 5543-58-8	warfarin
1		zidovudine
1		ziprasidone
1		zoledronic acid
1		zonisamide
1		ziv-aflibercept
2	72-55-9	1,1-Dichloro-2,2-bis(p-chloropheny)ethylene (DDE)
2	106-99-0	1,3-butadiene
2	111-77-3	2-(2-methoxyethoxy)ethanol
2	1746-01-6	2,3,7,8-Tetrachlorodibenzo-p-dioxin (TCDD)
2	121-14-2	2,4-dinitrotoluene
2	606-20-2	2,6-dinitrotoluene
2	2040-90-6	2-chloro-6-fluoro-phenol
2	88-72-2	2-nitrotoluene
2	101-80-4	4,4'-oxydianiline and its salts
2	84852-15-3 ; 104-40-5	4-nonylphenol, branched
2	71751-41-2	abamectin (combination of avermectin B1a and avermectin B1b)
2	34256-82-1	acetochlor
2	61-82-5	amitrole
2	1912-24-9	atrazine
2	71-43-2, 1076-43-3	benzene
2	1689-84-5	bromoxynil
2	1689-99-2	bromoxynil octanoate
2	94-26-8	butyl 4-hydroxybenzoate
2	7440-43-9	cadmium (non-pyrophoric) ;cadmium (pyrophoric)
2	63-25-2	carbaryl
2	143-50-0	chlordecone (kepone)
2	1333-82-0	chromium (vi) trioxide
2	120-32-1	clorofene
2	420-04-2	cyanamide
2	106-89-8	epichlorohydrin
2	106-93-4	ethylene dibromide
2	75-21-8	ethylene oxide
2	60168-88-9	fenarimol
2	900-95-8	fentin acetate

2	76-87-9	fentin hydroxide
2	151-67-7	halothane
2	680-31-9	hexamethylphosphoric triamide
2	22398-80-7	indium phosphide
2	1689-83-4	ioxynil
2	8018-01-7	mancozeb
2	12427-38-2	maneb
2	99-65-0	m-dinitrobenzene
2	137-42-8	metham sodium
2	9006-42-2	metiram
2	2385-85-5	mirex
2	NoCAS 038	mixture of 2,3,4,5-tetrachlorobiphenyl (PCB 61), 2,2',4,5,5'-octachlorobiphenyl (PCB 101) and 2,2',3,3',4,4',5,5'-octachlorobiphenyl (PCB 194)
2	2212-67-1	molinate
2	88671-89-0	myclobutanil
2	25154-52-3	nonylphenol
2	789-02-6	o,p'-DDT
2	556-67-2	octamethylcyclotetrasiloxane
2	50-29-3	p,p'-DDT = clofenotane
2	NoCAS 004	PBBs = Brominated Flame retardants = PBB (mixed group of 209 Congeners)
2	1336-36-3	PCB
2	NoCAS 039	PCB 104 (2,2',4,6,6'-pentachlorobiphenyl)
2	NoCAS 041	PCB 105 (2,3,3',4,4' -pentachlorobiphenyl)
2	NoCAS 092	PCB 114 (2,3,4,4',5-pentachlorobiphenyl)
2	31508-00-6	PCB 118 (2,3',4,4',5-pentachlorobiphenyl)
2	NoCAS 042	PCB 122 (2,3,3',4,5 -pentachlorobiphenyl)
2	NoCAS 037	PCB 126 (3,3',4,4',5-pentachlorobiphenyl)
2	38380-07-3	PCB 128 (2,2',3,3',4,4'-hexachlorobiphenyl)
2	35065-27-1	PCB 153 (2,2',4,4',5,5'-hexachlorobiphenyl)
2	32774-16-6	PCB 169 (3,3',4,4',5,5'-hexachlorobiphenyl)
2	37680-65-2	PCB 18 (2,2',5-trichlorobiphenyl)
2	55702-46-0	PCB 21 (2,3,4-trichlorobiphenyl)
2	7012-37-5	PCB 28 (2,4,4'-trichlorobiphenyl)
2	2437-79-8	PCB 47 (2,2',4,4'-tetrachlorobiphenyl)
2	35693-99-3	PCB 52 (2,2';5,5'-tetrachlorobiphenyl)
2	32598-13-3	PCB 77 (3,3',4,4'-tetrachlorobiphenyl)
2	NoCAS 036	PCB aroclor 1016
2	53469-21-9	PCB aroclor 1242
2	12672-29-6	PCB aroclor 1248
2	11097-69-1	PCB aroclor 1254
2	11096-82-5	PCB aroclor 1260 (clophen A60)
2	NoCAS 087	PCB138 2,2',3,4,4',5'-hexachlorobiphenyl
2	NoCAS 088	PCB180 2,2',3,4,4',5,5'-heptachlorobiphenyl
2	77-09-8	phenolphthalein
2	84-66-2	diethyl phthalate
2	26761-40-0	diisodecyl phthalate

2	94-13-3	propyl 4-hydroxybenzoate
2	2439-01-2	quinomethionate
2	10453-86-8	resmethrin
2	100-42-5	styrene
2	2155-70-6	tributyltin methacrylate
3	144177-62-8	(iR,S/i)-2-amino-3,3-dimethylbutaneamide
3	143322-57-0	(iR/i)-5-bromo-3-(1-methyl-2-pyrrolidinylmethyl)-1iH/i-indole
3	79815-20-6	(iS/i)-2,3-dihydro-1iH/i-indole-2-carboxylicacid
3	40722-80-3	(2-chloroethyl)(3-hydroxypropyl)ammoniumchloride
3	156145-66-3	(ethenylmethylsilylene)di[(4-methylpentan-2-one)oxime]
3	51229-78-8	icis/i-1-(3-chloroallyl)-3,5,7-triaza-1-azoniaadamantanechloride
3	149591-38-8	iN/i,iN/i'-dihexadecyl-iN/i,iN/i'-bis(2-hydroxyethyl)propanediamide
3	777891-21-1	iN/i-[2-(3-acetyl-5-nitrothiophen-2-ylazo)-5-diethylaminophenyl]acetamide
3		iO/i-hexyl-iN/i-ethoxycarbonylthiocarbamate
3	103122-66-3	iO/i-isobutyl-iN/i-ethoxycarbonylthiocarbamate
3	90657-55-9	itrans/i-4-cyclohexyl-L-prolinemonohydrochloride
3	13010-47-4	1-(2-Chloroethyl)-3-cyclohexyl-1-nitrosourea(CCNU)(Lomustine)
3	288-88-0	1,2,4-triazole
3	3194-55-6	1,2,5,6,9,10-hexabromocyclododecane
3	110-88-3	1,3,5-trioxan
3	2451-62-9	1,3,5-tris(oxiranylmethyl)-1,3,5-triazine-2,4,6(1iH/i,3iH/i,5iH/i)-trione
3	59653-74-6	1,3,5-tris-[(2iS/iand2iR/i)-2,3-epoxypropyl]-1,3,5-triazine-2,4,6-(1iH/i,3iH/i,5iH/i)-trione
3	55-98-1	1,4-Butanediol dimethanesulfonate (Busulfan)
3	123-91-1	1,4-dioxacyclohexane
3	93107-30-3	1-cyclopropyl-6,7-difluoro-1,4-dihydro-4-oxoquinoline-3-carboxylic acid
3	111-90-0	2-(2-ethoxyethoxy)ethanol
3	99610-72-7	2-(2-hydroxy-3,5-dinitroanilino)ethanol
3	5406-86-0	2-(4- i tert /i -butylphenyl)ethanol
3	1464-53-5	2,2'-bioxirane
3	96-13-9	2,3-dibromopropan-1-ol
3	602-01-7	2,3-dinitrotoluene
3	3033-77-0	2,3-epoxypropyltrimethylammonium chloride ...%
3	3397-62-4	2,4-diamino-6-chloro-s-triazine (DACT)
3	619-15-8	2,5-dinitrotoluene
3	110-13-4	2,5-hexanedione
3	784157-49-9	2-{}{4-(2-ammoniopropylamino)-6-[4-hydroxy-3-(5-methyl-2-methoxy-4-sulfamoylphenylazo)-2-sulfonatonaphth-7-ylamino]-1,3,5-triazin-2-ylamino}}-2-aminopropylformate
3	141-43-5	2-aminoethanol

3	79-07-2	2-chloracetamide
3	598-78-7	2-chloropropionicacid
3	637-92-3	2-ethoxy-2-methylpropane
3	149-57-5	2-ethylhexanoicacid
3	57583-35-4	2-ethylhexyl10-ethyl-4,4-dimethyl-7-oxo-8-oxa-3,5-dithia-4-stannatetradecanoate
3	57583-34-3	2-ethylhexyl10-ethyl-4-[[2-[(2-ethylhexyl)oxy]-2-oxoethyl]thio]-4-methyl-7-oxo-8-oxa-3,5-dithia-4-stannatetradecanoate
3		2-methyl-5-itert/i-butylthiophenol
3	87691-88-1	3-(piperazin-1-yl)-benzo[d]isothiazolehydrochloride
3	610-39-9	3,4-dinitrotoluene
3	618-85-9	3,5-dinitrotoluene
3	5146-66-7	3,7-dimethylocta-2,6-dienenitrile
3	302-97-6	3-oxoandrost-4-ene-17-ß-carboxylicacid
3	13595-25-0	4,4'-(1,3-phenylene-bis(1-methylethylidene))bis-phenol
3	159939-85-2	4-[(3-chlorophenyl)(1iH/i-imidazol-1-yl)methyl]-1,2-benzenediaminedihydrochloride
3	1671-49-4	4-mesyl-2-nitrotoluene
3	95-80-7	4-methyl-im/i-phenylenediamine
3	98-54-4	4-tert-butylphenol
3	100-40--3	4-vinylcyclohexene
3	115662-06-1	5,6,12,13-tetrachloroanthra(2,1,9-idef/i:6,5,10-id/i'ie/i'if/i')diisoquinoline-1,3,8,10(2iH/i,9iH/i)-tetrone
3	17630-75-0	5-chloro-1,3-dihydro-2 i H /i -indol-2-one
3	141-78-6	ethyl acetate
3	112-15-2	2-(2-ethoxyethoxy)ethyl acetate
3	123-86-4	N-butyl acetate
3	59-66-5	acetazolamide
3	546-88-3	acetohydroxamic acid
3	67-64-1	acetone
3	68515-49-1	1,2-benzenedicarboxylic acid, di-C9-11-branched alkyl esters, C10-rich
3	50-76-0	actinomycin D
3	68475-57-0	alkanes, C1-2
3	90622-55-2	alkanes, C1-4, C3-rich
3	68475-58-1	alkanes, C2-3
3	68475-59-2	alkanes, C3-4
3	68475-60-5	alkanes, C4-5
3	302-79-4	all-trans retinoic acid
3	106-92-3	allyl glycidyl ether
3	28981-97-7	Alprazolam
3	665-66-7	amantadine hydrochloride
3	39831-55-5	amikacin sulfate
3	125-84-8	aminoglutethimide
3		aminoglycosides
3	54-62-6	aminopterin
3	19774-82-4	amiodarone hydrochloride

3	33089-61-1	amitraz
3	14028-44-5	amoxapine
3		anabolic steroids
3	15375-21-0	androsta-1,4,9(11)-triene-3,17-dione
3		Angiotensin converting enzyme (ACE) inhibitors
3	117-37-3	Anisindione
3	90640-81-6	Anthracene oil, anthracene paste
3	91995-15-2	anthracene oil, anthracene paste, anthracene fraction
3	91995-16-3	anthracene oil, anthracene paste, carbazole fraction
3	91995-17-4	anthracene oil, anthracene paste, distn. lights
3	90640-82-7	anthracene oil, anthracene-low
3	68131-49-7	aromatichydrocarbons,Csub6-10/sub,acid-treated,neutralized
3	90989-41-6	aromatichydrocarbons,Csub6-10/sub,Csub8/sub-rich
3	68475-70-7	aromatichydrocarbons,Csub6-8/sub,naphtha-raffinatepyrolyzate-derived
3	93571-75-6	aromatichydrocarbons,Csub7-12/sub,Csub8/sub-rich
3	90989-42-7	aromatichydrocarbons,Csub7-8/sub,dealkylationproducts,distn.residues
3	90989-38-1	aromatichydrocarbons,Csub8/sub
3	91995-18-5	aromatichydrocarbons,Csub8/sub,catalyticreforming-derived
3	90989-39-2	aromatichydrocarbons,Csub8-10/sub
3	91995-20-9	aromatichydrocarbons,Csub8-9/sub,hydrocarbonresinpolymn.by-product
3	92062-36-7	aromatichydrocarbons,Csub9-12/sub,benzenedistn.
3	7440-38-2	arsenic (inorganic oxides)
3	1303-11-3	Indium arsenide
3	50-78-2	aspirin
3	29122-68-7	atenolol
3	34031-32-8	auranofin
3	65195-55-3	avermectin B1a (purity ≥80 %)
3		barbiturates
3	5534-09-8	beclomethasone dipropionate
3	82560-54-1	benfuracarb
3		benzodiazepines
3	65996-88-5	benzol forerunnings (coal)
3	5411-22-3	benzphetamine hydrochloride
3	23085-60-1	benzyl 2,4-dibromobutanoate
3	125051-32-3	bis(η sup 5 /sup -cyclopentadienyl)-bis(2,6-difluoro-3-[pyrrol-1-yl]-phenyl)titanium
3	154-93-8	bischloroethyl nitrosourea (BCNU) (carmustine)
3	53404-19-6	bromacil lithium salt
3	56634-95-8	bromoxynil heptanoate
3	143-81-7	Butabarbital sodium
3	106-97-8	butane (containing ≥ 0,1 % butadiene (203-450-8))
3	138164-12-2	butroxydim
3	513-78-0	cadmium carbonate

3	21041-95-2	cadmium hydroxide
3	10022-68-1, 10325-94-7	cadmium nitrate
3	1306-19-0	cadmium oxide (non-pyrophoric)
3	1306-23-6	cadmium sulphide
3	474-25-9	chenodiol
3	1620-21-9	chlorcyclizine hydrochloride
3	58-25-3	chlordiazepoxide
3	438-41-5	chlordiazepoxide hydrochloride
3	15545-48-9	chlorotoluron
3	10025-82-8	chlorure d'indium (forme micrometrique)
3	8001-54-5	chlorures de benzalkonium
3	14977-61-8	chromyl dichloride
3	81103-11-9	clarithromycin
3	25122-46-7	clobetasol propionate
3	50-36-2	cocaine
3		cocamidopropyl betaïne ; lexaine c
3	52-28-8	codeine phosphate
3		conjugated estrogens
3	21725-46-2	cyanazine
3	1134-23-2	cycloate
3	108-94-1	cyclohexanone
3	108-91-8	cyclohexylamine
3	120-92-3	cyclopentanone
3	6055-19-2	cyclophosphamide (hydrated)
3	101205-02-1	cycloxydim
3	13121-70-5	cyhexatin
3	57966-95-7	cymoxanil
3	17230-88-5	danazol
3	64-73-3	demeclocycline hydrochloride (internal use)
3	6190-65-4	des-ethyl atrazine (DEA)
3	1007-28-9	des-isopropyl atrazine (DIA)
3		diammonium 1-hydroxy-2-(4-(4-carboxyphenylazo)-2,5-dimethoxyphenylazo)-7-amino-3-naphthalenesulfonate
3	439-14-5	diazepam
3	364-98-7	diazoxide
3	79-43-6	dichloroacetic acid
3	97-23-4	dichlorophene
3	120-97-8	dichlorphenamide
3	51338-27-3	diclofop methyl
3	66-76-2	dicumarol
3	64-67-5	diethyl sulphate
3	22494-42-4	diflunisal
3	6190-39-2	dihydroergotamine mesylate
3	28553-12-0	di-isononyl phthalate(dinp)
3	33286-22-5	diltiazem hydrochloride
3	753-73-1	dimethyltin dichloride
3	149961-52-4	dimoxystrobin
3	25321-14-6	dinitrotoluene

3	117-84-0	di-n-octyl phthalate (DnOP)
3	75980-60-8	diphenyl(2,4,6-trimethylbenzoyl)phosphine oxide
3	57-41-0	Diphenylhydantoin (Phenytoin)
3	573-58-0	disodium 3,3'-[[1,1'-biphenyl]-4,4'-diylbis(azo)]bis(4-aminonaphthalene-1-sulphonate)
3	1937-37-7	disodium 4-amino-3-[[4'-[(2,4-diaminophenyl)azo][1,1'-biphenyl]-4-yl]azo]-5-hydroxy-6-(phenylazo)naphtalene-2,7-disulphonate
3	138-93-2	disodium cyanodithioimidocarbonate
3	101896-26-8	distillates (coal tar), benzole fraction, BTX-rich
3	121620-46-0	distillates (coal tar), benzole fraction, distn. residues
3	90640-87-2	distillates (coal tar), light oils, acid exts.; light oil extract residues, high boiling
3	90640-88-3	distillates (coal tar), light oils, alk. exts.
3	101794-90-5	Distillates (coal tar), light oils, neutral fraction; Light Oil Extract Residues, high boiling
3	84650-03-3	distillates (coal tar), light oils; carbolic Oil
3	91995-49-2	distillates (coal tar), naphthalene oil crystn. mother liquor
3	84650-04-4	distillates (coal tar), naphthalene oils
3	91995-48-1	distillates (coal tar), naphthalene oils, acid exts.
3	90640-89-4	distillates (coal tar), naphthalene oils, alk. exts.
3	101794-91-6	distillates (coal tar), naphthalene oils, indole-methylnaphthalene fraction
3	101896-27-9	distillates (coal tar), naphthalene oils, methylnaphthalene fraction
3	90640-90-7	distillates (coal tar), naphthalene oils, naphthalene-free, alk. exts.
3	84989-09-3	distillates (coal tar), naphthalene oils, naphthalene-low
3	91995-35-6	distillates (coal), coal tar-residual pyrolysis oils, naphthalene oils
3	85029-51-2	distillates (coal), coke-oven light oil, naphthalene cut
3	94114-52-0	distillates (coal), liq. solvent extn., primary
3	94114-53-1	distillates (coal), solvent extn., hydrocracked
3	94114-57-5	distillates (coal), solvent extn., hydrocracked hydrogenated middle
3	94114-56-4	distillates (coal), solvent extn., hydrocracked middle
3	91995-31-2	distillates (petroleum), alkene-alkyne manuf. pyrolysis oil, mixed with high-temp. coal tar, indene fraction
3	68477-34-9	distillates (petroleum), c sub 3-5 /sub , 2-methyl-2-butene-rich
3	93165-19-6	distillates (petroleum), c sub 6 /sub -rich
3	101316-56-7	distillates (petroleum), c sub 7-9 /sub , c sub 8 /sub -rich, hydrodesulfurized dearomatized
3	68477-35-0	distillates (petroleum), C3-6, piperylene-rich
3	68475-79-6	distillates (petroleum), catalytic reformed depentanizer
3	85116-58-1	distillates (petroleum), catalytic reformed hydrotreated light, C sub 8-12 /sub arom. fraction
3	68513-63-3	distillates (petroleum), catalytic reformed straight-run naphtha overheads

3	68477-89-4	distillates (petroleum), depentanizer overheads
3	91995-41-4	distillates (petroleum), heat-soaked steam-cracked naphtha, c sub 5 /sub -rich
3	67891-79-6	distillates (petroleum), heavy arom.
3	68410-98-0	distillates (petroleum), hydrotreated heavy naphtha, deisohexanizer overheads
3	68410-96-8	distillates (petroleum), hydrotreated middle, intermediate boiling
3	67891-80-9	distillates (petroleum), light arom.
3	68410-97-9	distillates (petroleum), light distillate hydrotreating process, low-boiling
3	68921-08-4	distillates (petroleum), light straight-run gasoline fractionation stabilizer overheads
3	68955-29-3	distillates (petroleum), light thermal cracked, debutanized arom.
3	91995-50-5	distillates (petroleum), naphtha steam cracking-derived, hydrotreated light arom.
3	91995-53-8	distillates (petroleum), naphtha steam cracking-derived, solvent-refined light hydrotreated
3	68921-09-5	distillates (petroleum), naphtha unifiner stripper
3	68425-29-6	distillates (petroleum), naphtha-raffinate pyrolyzate-derived, gasoline-blending
3	68477-50-9	distillates (petroleum), polymd. steam-cracked petroleum distillates, c sub 5-12 /sub fraction
3	68477-55-4	distillates (petroleum), steam-cracked, c sub 5-10 /sub fraction, mixed with light steam-cracked petroleum naphtha c sub 5 /sub fraction
3	68477-53-2	distillates (petroleum), steam-cracked, c sub 5-12 /sub fraction
3	95009-23-7	distillates (petroleum), steam-cracked, c sub 8-12 /sub fraction, polymd., distn. lights
3	68410-05-9	Distillates (petroleum), straight-run light
3	68603-00-9	Distillates (petroleum), thermal cracked naphtha and gas oil
3	68603-01-0	Distillates (petroleum), thermal cracked naphtha and gas oil, C sub 5 /sub -dimer-contg.
3	68603-03-2	Distillates (petroleum), thermal cracked naphtha and gas oil, extractive
3	1314-62-1	divanadium pentaoxide
3	1593-77-7	dodemorph
3	31717-87-0	dodemorph acetate
3	564-25-0	Doxycycline (internal use)
3	94088-85-4	doxycycline calcium (internal use)
3	24390-14-5	doxycycline hyclate (internal use)
3	17086-28-1	doxycycline monohydrate (internal use)
3	72-20-8	endrin
3		environmental tobacco smoke (ets)
3	379-79-3	ergotamine tartrate
3	8006-64-2	essence de terebenthine

3	536-33-4	ethionamide
3	759-94-4	ethyl dipropylthiocarbamate
3	151-56-4	ethyleneimine
3	41340-25-4	etodolac
3	54350-48-0	etretinate
3	84989-12-8	Extract oils (coal), acidic, tar-base free
3	122070-80-8	extract oils (coal), coal tar residual pyrolysis oils, naphthalene oil, distn. residues
3	91995-66-3	extract oils (coal), coal tar-residual pyrolysis oils, naphthalene oil, redistillate
3	122070-79-5	extract oils (coal), coal tar-residual pyrolysis oils, naphthalene oils
3	90640-99-6	extract oils (coal), light oil
3	90641-00-2	extract oils (coal), naphthalene oils
3	65996-86-3	extract oils (coal), tar base
3	68937-63-3	extract oils (coal), tar base, collidine fraction
3	101316-63-6	extract residues (coal tar), benzole fraction alk., acid ext.
3	93821-38-6	extract residues (coal), benzole fraction acid
3	91995-61-8	extract residues (coal), benzole fraction alk., acid ext.
3	90641-01-3	extract residues (coal), light oil alk., acid ext.
3	101316-62-5	extract residues (coal), light oil alk., acid ext., indene fraction
3	90641-02-4	extract residues (coal), light oil alk., distn. overheads
3	90641-03-5	extract residues (coal), light oil alk., indene naphtha fraction
3	122384-78-5	extract residues (coal), low temp. coal atar alk.
3	90641-04-6	extract residues (coal), naphthalene oil alk., distn. overheads
3	90641-05-7	extract residues (coal), naphthalene oil alk., distn. residues
3	121620-47-1	extract residues (coal), naphthalene oil, alk.
3	121620-48-2	extract residues (coal), naphthalene oil, alk., naphthalene-low
3	65996-87-4	extract residues (coal), tar oil alk.
3	90641-06-8	extract residues (coal), tar oil alk., carbonated, limed
3	73665-18-6	extract residues (coal), tar oil alk., naphthalene distn. residues
3	97926-43-7	extracts (petroleum) heavy naphtha solvent, clay-treated
3	91995-68-5	extracts (petroleum), catalytic reformed light naphtha solvent
3	68477-61-2	extracts (petroleum), cold-acid, c sub 4-6 /sub
3	65996-83-0	extracts, coal tar oil alk.
3	66441-23-4	fenoxaprop ethyl
3	67564-91-4	fenpropimorph
3	121181-53-1	filgrastim
3	79241-46-6	fluazifop-p-butyl
3	79622-59-6	fluazinam
3	3385-03-3	flunisolide
3	1172-18-5	flurazepam hydrochloride
3	5104-49-4	flurbiprofen

3	80474-14-2	fluticasone propionate
3	69409-94-5	fluvalinate
3	68476-26-6	fuel gases
3	68476-29-9	fuel gases, crude oil of distillates
3	107910-75-8	ganciclovir sodium
3	68606-27-9	gases (petroleum), alkylation feed
3	68477-65-6	gases (petroleum), amine system feed
3	68477-66-7	gases (petroleum), benzene unit hydrodesulfurizer off
3	68602-82-4	gases (petroleum), benzene unit hydrotreater depentanizer overheads
3	68477-67-8	gases (petroleum), benzene unit recycle, hydrogen-rich
3	68477-68-9	gases (petroleum), blend oil, hydrogen-nitrogen-rich
3	68477-69-0	gases (petroleum), butane splitter overheads
3	68602-83-5	gases (petroleum), c1-5, wet
3	68477-70-3	gases (petroleum), c2-3-
3	68783-65-3	gases (petroleum), c2-4, sweetened
3	68477-84-9	gases (petroleum), C2-return stream
3	68131-75-9	gases (petroleum), C3-4
3	68477-33-8	gases (petroleum), C3-4, isobutane-rich
3	68477-83-8	gases (petroleum), C3-5 olefinic-paraffinic alkylation feed
3	68477-85-0	gases (petroleum), C4-rich
3	68477-81-6	gases (petroleum), C6-8 catalytic reformer
3	68477-80-5	gases (petroleum), C6-8 catalytic reformer recycle
3	68477-82-7	gases (petroleum), C6-8 catalytic reformer recycle, hydrogen-rich
3	68952-76-1	gases (petroleum), catalytic cracked naphtha debutanizer
3	68477-73-6	gases (petroleum), catalytic cracked naphtha depropanizer overhead, c3-rich acid-free
3	68409-99-4	gases (petroleum), catalytic cracked overheads
3	68477-74-7	gases (petroleum), catalytic cracker
3	68477-75-8	gases (petroleum), catalytic cracker, c1-5-rich
3	68783-64-2	gases (petroleum), catalytic cracking
3	68477-76-9	gases (petroleum), catalytic polymd. naphtha stabilizer overhead, c2-4-rich
3	68477-77-0	gases (petroleum), catalytic reformed naphtha stripper overheads
3	68513-14-4	gases (petroleum), catalytic reformed straight-run naphtha stabilizer overheads
3	68477-79-2	gases (petroleum), catalytic reformer, c1-4-rich
3	68477-71-4	gases (petroleum), catalytic-cracked gas oil depropanizer bottoms, C4-rich acid-free
3	68477-72-5	gases (petroleum), catalytic-cracked naphtha debutanizer bottoms, c3-5-rich
3	68989-88-8	gases (petroleum), crude distn. And catalytic cracking
3	68918-99-0	gases (petroleum), crude oil fractionation off
3	68477-86-1	gases (petroleum), deethanizer overheads
3	68919-00-6	gases (petroleum), dehexanizer off
3	68477-87-2	gases (petroleum), deisobutanizer tower overheads

3	68606-34-8	gases (petroleum), depropanizer bottoms fractionation off
3	68477-90-7	gases (petroleum), depropanizer dry, propene-rich
3	68477-91-8	gases (petroleum), depropanizer overheads
3	68919-01-7	gases (petroleum), distillate unifiner desulfurization stripper off
3	68477-92-9	gases (petroleum), dry sour, gas-concn.-unit-off
3	68919-02-8	gases (petroleum), fluidized catalytic cracker fractionation off
3	68919-03-9	gases (petroleum), fluidized catalytic cracker scrubbing secondary absorber off
3	68919-20-0	gases (petroleum), fluidized catalytic cracker splitter overheads
3	68513-15-5	gases (petroleum), full-range straight-run naphtha dehexanizer off
3	68477-93-0	gases (petroleum), gas concn. reabsorber distn.
3	92045-15-3	gases (petroleum), gas oil diethanolamine scrubber off
3	92045-16-4	gases (petroleum), gas oil hydrodesulfurization effluent
3	92045-17-5	gases (petroleum), gas oil hydrodesulfurization purge
3	68477-94-1	gases (petroleum), gas recovery plant depropanizer overheads
3	68477-95-2	gases (petroleum), girbotol unit feed
3	68919-04-0	gases (petroleum), heavy distillate hydrotreater desulfurization stripper off
3	68513-16-6	gases (petroleum), hydrocracking depropanizer off, hydrocarbon-rich
3	68783-06-2	gases (petroleum), hydrocracking low-pressure separator
3	68477-96-3	gases (petroleum), hydrogen absorber off
3	92045-18-6	gases (petroleum), hydrogenator effluent flash drum off
3	68477-97-4	gases (petroleum), hydrogen-rich
3	68911-58-0	gases (petroleum), hydrotreated sour kerosine depentanizer stabilizer off
3	68911-59-1	gases (petroleum), hydrotreated sour kerosine flash drum
3	68477-98-5	gases (petroleum), hydrotreater blend oil recycle, hydrogen-nitrogen-rich
3	68477-99-6	gases (petroleum), isomerized naphtha fractionator, c4-rich, hydrogen sulfide-free
3	68919-05-1	gases (petroleum), light straight run gasoline fractionation stabilizer off
3	68513-17-7	gases (petroleum), light straight-run naphtha stabilizer off
3	92045-19-7	gases (petroleum), naphtha steam cracking high-pressure residual
3	68919-06-2	gases (petroleum), naphtha unifiner desulfurization stripper off
3	68527-15-1	gases (petroleum), oil refinery gas distn. off
3	68814-90-4	gases (petroleum), platformer products separator off
3	68919-07-3	gases (petroleum), platformer stabilizer off, light ends fractionation
3	68919-08-4	gases (petroleum), preflash tower off, crude distn.
3	68478-00-2	gases (petroleum), recycle, hydrogen-rich
3	68814-67-5	gases (petroleum), refinery

3	68783-07-3	gases (petroleum), refinery blend
3	68513-18-8	gases (petroleum), reformer effluent high-pressure flash drum off
3	68513-19-9	gases (petroleum), reformer effluent low-pressure flash drum off
3	68478-01-3	gases (petroleum), reformer make-up, hydrogen-rich
3	68478-02-4	gases (petroleum), reforming hydrotreater
3	68478-04-6	gases (petroleum), reforming hydrotreater make-up, hydrogen-rich
3	68478-03-5	gases (petroleum), reforming hydrotreater, hydrogen-methane-rich
3	92045-20-0	gases (petroleum), residue visbaking off
3	68602-84-6	gases (petroleum), secondary absorber off, fluidized catalytic cracker overheads fractionator
3	68955-33-9	gases (petroleum), sponge absorber off, fluidized catalytic cracker and gas oil desulfurizer overhead fractionation
3	92045-22-2	gases (petroleum), steam-cracker c3-rich
3	68955-34-0	gases (petroleum), straight-run naphtha catalytic reformer stabilizer overhead
3	68919-09-5	gases (petroleum), straight-run naphtha catalytic reforming off
3	68919-10-8	gases (petroleum), straight-run stabilizer off
3	68919-11-9	gases (petroleum), tar stripper off
3	68478-05-7	gases (petroleum), thermal cracking distn.
3	68919-12-0	gases (petroleum), unifiner stripper off
3	68955-28-2	gases (petroleum, light steam-cracked, butadiene conc.
3	86290-81-5	gasoline
3	93572-29-3	gasoline, c sub 5-11 /sub , high-octane stabilised reformed
3	8006-61-9	gasoline, natural
3	68606-10-0	gasoline, pyrolysis, debutanizer bottoms
3	94114-03-1	gasoline, pyrolysis, hydrogenated
3	68606-11-1	gasoline, straight-run, topping-plant
3	68514-15-8	gasoline, vapor-recovery
3	25812-30-0	gemfibrozil
3	23092-17-3	halazepam
3	66852-54-8	halobetasol propionate
3	52-86-8	haloperidol
3	76-44-8	heptachlor
3	25637-99-4	hexabromocyclododecane
3	684-16-2	hexafluoroacetone
3	67485-29-4	hydramethylnon
3	100801-63-6	hydrocarbon oils, arom., mixed with polyethylene and polypropylene, pyrolyzed, light oil fraction
3	100801-65-8	hydrocarbon oils, arom., mixed with polyethylene, pyrolyzed, light oil fraction
3	100801-66-9	hydrocarbon oils, arom., mixed with polystyrene, pyrolyzed, light oil fraction
3	68476-50-6	hydrocarbons, c sub ≥5 /sub , c sub 5-6 /sub -rich

3	68476-47-1	hydrocarbons, c sub 2-6 /sub , c sub 6-8 /sub catalytic reformer
3	68476-46-0	hydrocarbons, c sub 3-11 /sub , catalytic cracker distillates
3	102110-14-5	hydrocarbons, c sub 3-6 /sub , c sub 5 /sub -rich, steam-cracked naphtha
3	92045-63-1	hydrocarbons, c sub 4-11 /sub , naphtha-cracking, arom.-free
3	92045-61-9	hydrocarbons, c sub 4-12 /sub , naphtha-cracking, hydrotreated
3	91995-38-9	hydrocarbons, c sub 4-6 /sub , depentanizer lights, arom. hydrotreater
3	68476-55-1	hydrocarbons, c sub 5 /sub -rich
3	102110-15-6	hydrocarbons, c sub 5 /sub -rich, dicyclopentadiene-contg.
3	93572-36-2	hydrocarbons, c sub 5-11 /sub , nonaroms.-rich, reforming light fraction
3	101316-67-0	hydrocarbons, c sub 6 /sub -rich, hydrotreated light naphtha distillates, solvent-refined
3	93763-33-8	hydrocarbons, c sub 6-11 /sub , hydrotreated, dearomatized
3	92045-64-2	hydrocarbons, c sub 6-7 /sub , naphtha-cracking, solvent-refined
3	101316-66-9	hydrocarbons, c sub 6-8 /sub , hydrogenated sorption-dearomatized, toluene raffination
3	93572-35-1	hydrocarbons, c sub 7-12 /sub , c sub ≥9 /sub -arom.-rich, reforming heavy fraction
3	92045-62-0	hydrocarbons, c sub 8-11 /sub , naphtha-cracking, toluene cut
3	101794-97-2	hydrocarbons, c sub 8-12 /sub , catalytic cracker distillates
3	101896-28-0	hydrocarbons, c sub 8-12 /sub , catalytic cracking, chem. neutralized, sweetened
3	92128-94-4	hydrocarbons, c sub 8-12 /sub , catalytic-cracking, chem. neutralized
3	93763-34-9	hydrocarbons, c sub 9-12 /sub , hydrotreated, dearomatized
3	68527-16-2	hydrocarbons, C1-3
3	68514-31-8	hydrocarbons, C1-4
3	68527-19-5	hydrocarbons, C1-4, debutanizer fraction
3	68514-36-3	Hydrocarbons, C1-4, sweetened
3	68606-25-7	Hydrocarbons, C2-4
3	68476-49-3	Hydrocarbons, C2-4, C3-rich
3	68606-26-8	Hydrocarbons, C3
3	68476-40-4	Hydrocarbons, C3-4
3	68512-91-4	Hydrocarbons, C3-4-rich, petroleum distillate
3	87741-01-3	Hydrocarbons, C4
3	95465-89-7	Hydrocarbons, C4, 1,3-butadiene- and isobutene-free
3	92045-23-3	Hydrocarbons, C4, steam-cracker distillate
3	68476-42-6	Hydrocarbons, C4-5

3	92045-55-1	Hydrocarbons, hydrotreated light naphtha distillates, solvent-refined
3		Hydrogen cyanide (HCN) and cyanide salts (CN salts)
3	57852-57-0	Idarubicin hydrochloride
3	10043-66-0	Iodine-131
3	3861-47-0	ioxynil octanoate
3	75-28-5	isobutane (containing ≥ 0,1 % butadiene (203-450-8))
3	26675-46-7	Isoflurane
3	4759-48-2	Isotretinoin
3	141112-29-0	isoxaflutole
3	59-92-7	Levodopa
3	797-63-7	Levonorgestrel implants
3	65996-78-3	Light oil (coal), coke-oven
3	90641-11-5	Light oil (coal), semi-coking process
3	8032-32-4	Ligroine
3	554-13-2	Lithium carbonate
3	919-16-4	Lithium citrate
3	846-49-1	Lorazepam
3	75330-75-5	Lovastatin
3	569-64-2	malachite green hydrochloride
3	2437-29-8	malachite green oxalate
3	31431-39-7	Mebendazole
3	57-53-4	Meprobamate
3	7487-94-7	mercury dichloride
3	125116-23-6	metconazole
3	3963-95-9	Methacycline hydrochloride
3	20354-26-1	Methazole
3	15475-56-6	Methotrexate sodium
3	77402-05-2	methyl acrylamidoglycolate (containing ≥ 0,1 % acrylamide)
3	77402-03-0	methyl acrylamidomethoxyacetate (containing ≥ 0,1 % acrylamid)
3	74-87-3	Methyl chloride
3	108-10-1	methyl isobutyl ketone (MIBK)
3		methyl-phenylene diamine
3	59467-96-8	Midazolam hydrochloride
3	13614-98-7	minocycline hydrochloride (internal use)
3	142-59-6	nabam
3	8030-30-6	naphtha
3	90641-12-6	naphtha (coal), distn. residues
3	94114-54-2	naphtha (coal), solvent extn., hydrocracked
3	64742-15-0	naphtha (petroleum), acid-treated
3	68603-08-7	naphtha (petroleum), arom.-contg.
3	92045-49-3	naphtha (petroleum), c sub 4-12 /sub , butane-alkylate, isooctane-rich
3	68783-09-5	naphtha (petroleum), catalytic cracked light distd.
3	64742-66-1	naphtha (petroleum), catalytic dewaxed
3	68955-35-1	naphtha (petroleum), catalytic reformed

3	85116-59-2	naphtha (petroleum), catalytic reformed light, arom.-free fraction
3	64742-22-9	naphtha (petroleum), chemically neutralized heavy
3	64742-23-0	naphtha (petroleum), chemically neutralized light
3	68527-21-9	naphtha (petroleum), clay-treated full-range straight-run
3	68527-22-0	naphtha (petroleum), clay-treated light straight-run
3	64741-64-6	naphtha (petroleum), full-range alkylate
3	68527-27-5	naphtha (petroleum), full-range alkylate, butane-contg.
3	68513-02-0	naphtha (petroleum), full-range coker
3	68919-37-9	naphtha (petroleum), full-range reformed
3	64741-42-0	naphtha (petroleum), full-range straight-run
3	64741-65-7	naphtha (petroleum), heavy alkylate
3	64741-54-4	naphtha (petroleum), heavy catalytic cracked
3	92045-50-6	naphtha (petroleum), heavy catalytic cracked, sweetened
3	64741-68-0	naphtha (petroleum), heavy catalytic reformed
3	64741-78-2	naphtha (petroleum), heavy hydrocracked
3	92045-51-7	naphtha (petroleum), heavy steam-cracked, hydrogenated
3	101631-20-3	naphtha (petroleum), heavy straight run, arom.-contg.
3	64741-41-9	naphtha (petroleum), heavy straight-run
3	64741-83-9	naphtha (petroleum), heavy thermal cracked
3	101316-76-1	naphtha (petroleum), hydrodesulfurised full-range coker
3	92045-52-8	naphtha (petroleum), hydrodesulfurized full-range
3	64742-73-0	naphtha (petroleum), hydrodesulfurized light
3	92045-53-9	naphtha (petroleum), hydrodesulfurized light, dearomatized
3	85116-60-5	naphtha (petroleum), hydrodesulfurized thermal cracked light
3	64742-82-1	naphtha (petroleum), hydrodesulphurized heavy
3	64742-48-9	naphtha (petroleum), hydrotreated heavy
3	64742-49-0	naphtha (petroleum), hydrotreated light
3	92045-57-3	naphtha (petroleum), hydrotreated light steam-cracked
3	85116-61-6	naphtha (petroleum), hydrotreated light, cycloalkane-contg.
3	64741-70-4	naphtha (petroleum), isomerization
3	92045-58-4	naphtha (petroleum), isomerization, c sub 6 /sub - fraction
3	64741-66-8	naphtha (petroleum), light alkylate
3	64741-55-5	naphtha (petroleum), light catalytic cracked
3	92045-59-5	naphtha (petroleum), light catalytic cracked sweetened
3	64741-63-5	naphtha (petroleum), light catalytic reformed
3	68513-03-1	naphtha (petroleum), light catalytic reformed, arom.-free
3	92201-97-3	naphtha (petroleum), light heat-soaked, steam-cracked
3	64741-69-1	naphtha (petroleum), light hydrocracked
3	64742-83-2	naphtha (petroleum), light steam-cracked
3	68527-23-1	naphtha (petroleum), light steam-cracked arom.
3	68527-26-4	naphtha (petroleum), light steam-cracked, debenzenized
3	98219-46-6	naphtha (petroleum), light steam-cracked, debenzenized, thermally treated

3	93165-55-0	naphtha (petroleum), light steam-cracked, hydrogenated
3	98219-47-7	naphtha (petroleum), light steam-cracked, thermally treated
3	64741-46-4	naphtha (petroleum), light straight-run
3	64741-74-8	naphtha (petroleum), light thermal cracked
3	92045-65-3	naphtha (petroleum), light thermal cracked, sweetened
3	92045-60-8	naphtha (petroleum), light, c sub 5 /sub -rich, sweetened
3	68783-66-4	naphtha (petroleum), light, sweetened
3	64741-92-0	naphtha (petroleum), solvent-refined heavy
3	64741-84-0	naphtha (petroleum), solvent-refined light
3	68516-20-1	naphtha (petroleum), steam-cracked middle arom.
3	64741-87-3	naphtha (petroleum), sweetened
3	101795-01-1	naphtha (petroleum), sweetened light
3	68783-12-0	naphtha (petroleum), unsweetened
3	64741-48-6	natural gas (petroleum), raw liq. mix
3	68919-39-1	natural gas condensates
3	64741-47-5	natural gas condensates (petroleum)
3	1405-10-3	neomycin sulfate (internal use)
3	56391-57-2	netilmicin sulfate
3	110-54-3	n-hexane
3	54-11-5	nicotine
3	21829-25-4	nifedipine
3	66085-59-4	nimodipine
3	1929-82-4	nitrapyrin
3	67-20-9	nitrofurantoin
3	51-75-2	nitrogen mustard (mechlorethamine)
3	55-86-7	nitrogen mustard hydrochloride (mechlorethamine hydrochloride)
3	68-22-4 ; 57-63-6 ; 72-33-3	norethisterone (norethindrone) ; ethinyl estradiol ; mestranol
3	51-98-9	norethisterone acetate (norethindrone acetate)
3	6533-00-2	norgestrel
3	71-23-8	n-propanol
3	528-29-0	o-dinitrobenzene
3	39807-15-3	oxadiargyl
3	19666-30-9	oxadiazon
3	604-75-1	oxazepam
3	301-12-2	oxydemeton methyl
3	434-07-1	oxymetholone
3	79-57-2	oxytetracycline (internal use)
3	2058-46-0	oxytetracycline hydrochloride (internal use)
3	115-67-3	paramethadione
3	100-25-4	p-dinitrobenzene
3	66246-88-6	penconazole
3	52-67-5	penicillamine
3	57-33-0	pentobarbital sodium
3	94-36-0	peroxyde de dibenzoyle
3	68476-85-7	petroleum gases, liquefied

3	68476-86-8	petroleum gases, liquefied, sweetened
3	92045-80-2	petroleum gases, liquefied, sweetened, c4 fraction
3	68514-79-4	petroleum products, hydrofiner-powerformer reformates
3	68607-11-4	petroleum products, refinery gases
3	63-98-9	phenacemide
3	84988-93-2	phenols, ammonia liquor ext.
3	91079-47-9	phenols, c sub 9-11 /sub
3	435-97-2	phenprocoumon
3	638-21-1	phenylphosphine
3	14816-18-3	phoxim
3	53306-54-0	phtalate de di-2-propylheptyle
3	68515-49-1	diisodecyl phthalate
3	131-16-8	dipropyl phthalate
3	2062-78-4	pimozide
3	110-85-0	piperazine
3	142-64-3	piperazine dihydrochloride
3	6094-40-2	piperazine hydrochloride
3	1951-97-9	piperazine phosphate
3	18378-89-7	Plicamycin
3		polybrominated biphenyls
3		polychlorinated biphenyls
3	7789-00-6	potassium chromate
3	128-03-0	potassium dimethyldithiocarbamate
3	81131-70-6	pravastatin sodium
3	125-02-0	prednisolone sodium phosphate
3	139001-49-3	profoxydim
3	2312-35-8	Propargite
3	139-40-2	Propazine
3	75-56-9	propylene oxide
3	2122-19-2	propylenethiourea
3	51-52-5	propylthiouracil
3	68391-11-7	pyridine, alkyl derivs.
3	58-14-0	pyrimethamine
3	36735-22-5	quazepam
3	76578-14-8	quizalofop-ethyl
3	68410-71-9	raffinates (petroleum), catalytic reformer ethylene glycol-water countercurrent exts.
3	68425-35-4	raffinates (petroleum), reformer, lurgi unit-sepd.
3	97722-19-5	raffinates (petroleum), steam-cracked C4 fraction cuprous ammonium acetate extn., C3-5 and C3-5 unsatd., butadiene-free
3		reaction mass of: 4,7-bis(mercaptomethyl)-3,6,9-trithia-1,11-undecanedithiol
3	163879-69-4	reaction mass of: 5-[(4-[(7-amino-1-hydroxy-3-sulfo-2-naphthyl)azo]-2,5-diethoxyphenyl)azo]-2-[(3-phosphonophenyl)azo]benzoic acid

3		reaction mass of: Ca salicylates (branched C sub 10-14 /sub and C sub 18-30 /sub alkylated)
3		reaction mass of: dimethyl (2-(hydroxymethylcarbamoyl)ethyl)phosphonate
3		reaction mass of: triammonium 6-amino-3-((2,5-diethoxy-4-(3-phosphonophenyl)azo)phenyl)azo-4-hydroxy-2-naphthalenesulfonate
3	68478-16-0	residual oils (petroleum), deisobutanizer tower
3	92061-92-2	residues (coal tar), anthracene oil distn.
3	68513-66-6	residues (petroleum), alkylation splitter, c4-rich
3	68478-12-6	residues (petroleum), butane splitter bottoms
3	68478-15-9	residues (petroleum), c sub 6-8 /sub catalytic reformer
3	102110-55-4	residues (petroleum), steam-cracked light, arom.
3		retinol/retinyl esters, when in daily dosages in excess of 10,000 iu, or 3,000 retinol equivalents
3	13292-46-1	rifampin
3		salts of bromoxynil
3		salts of ioxynil
3	309-43-3	Secobarbital sodium
3	114466-38-5	Sermorelin acetate
3	130328-20-0	silver zinc zeolite (zeolite, lta framework type, surface-modified with silver and zinc ions)
3	122-34-9	simazine
3	128-04-1	sodium dimethyldithiocarbamate
3	62-74-8	sodium fluoroacetate
3	65996-79-4	solvent naphtha (coal)
3	85536-19-2	solvent naphtha (coal), coumarone-styrene contg.
3	85536-17-0	solvent naphtha (coal), light
3	85536-20-5	solvent naphtha (coal), xylene-styrene cut
3	92062-15-2	solvent naphtha (petroleum), hydrotreated light naphthenic
3	64742-89-8	solvent naphtha (petroleum), light aliph.
3	64742-95-6	solvent naphtha (petroleum), light arom.
3	68512-78-7	solvent naphtha (petroleum), light arom., hydrotreated
3	203313-25-1	spirotetramat
3	118134-30-8	spiroxamine
3	8052-41-3	stoddard solvent
3	3810-74-0	streptomycin sulfate
3	99105-77-8	sulcotrione
3	599-79-1	sulfasalazine (salicylazosulfapyridine)
3	7446-09-5	sulfur dioxide
3	38194-50-2	Sulindac
3	68478-21-7	tail gas (petroleum), catalytic cracked clarified oil and thermal cracked vacuum residue fractionation reflux drum
3	68307-98-2	tail gas (petroleum), catalytic cracked distillate and catalytic cracked naphtha fractionation absorber
3	68952-77-2	tail gas (petroleum), catalytic cracked distillate and naphtha stabilizer

3	68478-22-8	tail gas (petroleum), catalytic cracked naphtha stabilization absorber
3	68478-25-1	tail gas (petroleum), catalytic cracker refractionation absorber
3	68478-24-0	tail gas (petroleum), catalytic cracker, catalytic reformer and hydrodesulfurizer combined fractionater
3	68952-79-4	tail gas (petroleum), catalytic hydrodesulfurized naphtha separator
3	68307-99-3	tail gas (petroleum), catalytic polymn. naphtha fractionation stabilizer
3	68478-26-2	tail gas (petroleum), catalytic reformed naphtha fractionation stabilizer
3	68308-00-9	tail gas (petroleum), catalytic reformed naphtha fractionation stabilizer, hydrogen sulfide-free
3	68478-27-3	tail gas (petroleum), catalytic reformed naphtha separator
3	68478-28-4	tail gas (petroleum), catalytic reformed naphtha stabilizer
3	68478-29-5	tail gas (petroleum), cracked distillate hydrotreater separator
3	68308-01-0	tail gas (petroleum), cracked distillate hydrotreater stripper
3	68308-03-2	tail gas (petroleum), gas oil catalytic cracking absorber
3	68308-04-3	tail gas (petroleum), gas recovery plant
3	68308-05-4	tail gas (petroleum), gas recovery plant deethanizer
3	68308-06-5	tail gas (petroleum), hydrodesulfurized distillate and hydrodesulfurized naphtha fractionator, acid-free
3	68478-30-8	tail gas (petroleum), hydrodesulfurized straight-run naphtha separator
3	68308-07-6	tail gas (petroleum), hydrodesulfurized vacuum gas oil stripper, hydrogen sulfide-free
3	68308-08-7	tail gas (petroleum), isomerized naphtha fractionation stabilizer
3	68308-09-8	tail gas (petroleum), light straight-run naphtha stabilizer, hydrogen sulfide-free
3	68308-11-2	tail gas (petroleum), propane-propylene alkylation feed prep deethanizer
3	68478-32-0	tail gas (petroleum), saturate gas plant mixed stream, c4-rich
3	68478-33-1	tail gas (petroleum), saturate gas recovery plant, c1-2-rich
3	68308-10-1	tail gas (petroleum), straight-run distillate hydrodesulfurizer, hydrogen sulfide-free
3	68952-80-7	tail gas (petroleum), straight-run naphtha hydrodesulfurizer
3	68952-82-9	tail gas (petroleum), thermal cracked hydrocarbon fractionation stabilizer, petroleum coking
3	68952-81-8	tail gas (petroleum), thermal-cracked distillate, gas oil and naphtha absorber
3	68308-12-3	tail gas (petroleum), vacuum gas oil hydrodesulfurizer, hydrogen sulfide-free
3	68478-34-2	tail gas (petroleum), vacuum residues thermal cracker
3	84989-07-1	tar acids, 3,5-xylenol fraction

3	92062-22-1	tar acids, brown-coal gasification
3	94114-29-1	tar acids, brown-coal, c sub 2 /sub -alkylphenol fraction
3	101316-86-3	tar acids, brown-coal, crude
3	65996-85-2	tar acids, coal, crude
3	92062-26-5	tar acids, cresylic
3	68555-24-8	tar acids, cresylic, residues
3	68815-21-4	tar acids, cresylic, sodium salts, caustic solns.
3	96690-55-0	tar acids, distn. residues
3	84989-03-7	tar acids, ethylphenol fraction
3	84989-04-8	tar acids, methylphenol fraction
3	84989-05-9	tar acids, polyalkylphenol fraction
3	68477-23-6	tar acids, residues, distillates, first-cut
3	84989-06-0	tar acids, xylenol fraction
3	92062-27-6	tar bases, coal, aniline fraction
3	92062-28-7	tar bases, coal, collidine fraction
3	65996-84-1	tar bases, coal, crude
3	92062-29-8	tar bases, coal, distn. residues
3	91082-52-9	tar bases, coal, lutidine fraction
3	92062-33-4	tar bases, coal, picoline fraction
3	70321-67-4	tar bases, coal, quinoline derivs. fraction
3	91082-53-0	tar bases, coal, toluidine fraction
3	68513-87-1	tar bases, quinoline derivs.
3	94114-40-6	tar oils, brown-coal
3	65996-82-9	tar oils, coal
3	101316-87-4	tar oils, coal, low-temp.
3	107534-96-3	tebuconazole
3	335104-84-2	tembotrione
3	149979-41-9	tepraloxydim
3	5902-51-2	terbacil
3	58-20-8	testosterone cypionate
3	315-37-7	testosterone enanthate
3	127-18-4	tetrachloroethylene
3	60-54-8	tetracycline (internal use)
3	64-75-5	tetracycline hydrochloride (internal use)
3	7226-23-5	tetrahydro-1,3-dimethyl-1 i h /i -pyrimidin-2-one
3	2602-46-2	tetrasodium 3,3'-[[1,1'-biphenyl]-4,4'-diylbis(azo)]bis[5-amino-4-hydroxynaphthalene-2,7-disulphonate]
3	23564-05-8	thiophanate methyl
3		tobacco smoke (primary)
3	49842-07-1	tobramycin sulfate
3	95-70-5	2-methyl-p-phenylenediamine
3	96314-26-0	trans-4-phenyl-L-proline
3	43121-43-3	triadimefon
3	221354-37-6	triammonium 4-[4-[7-(4-carboxylatoanilino)-1-hydroxy-3-sulfonato-2-naphthylazo]-2,5-dimethoxyphenylazo]benzoate
3	28911-01-5	triazolam
3	79-01-6	trichloroethylene

3	993-16-8	trichloromethylstannane
3	38260-01-4	trientine hydrochloride
3	26644-46-2	triforine
3	13647-35-3	trilostane
3	127-48-0	trimethadione
3	82952-64-5	trimetrexate glucuronate
3	51-79-6	urethane (ethyl carbamate)
3	97048-13-0	urofollitropin
3	20108-78-5	valinamide
3	106-87-6	vinyl cyclohexene dioxide (4-vinyl-1-cyclohexene diepoxide)
3	64742-48-9 ; 64742-88-7 ; 64742-82-1 ;8052-41-3	white-spirit
3	111406-87-2	zileuton
3	5216-25-1	A, α,α,4-tetrachlorotoluene
3	25383-07-7	α-phenylethylammonium (-)-(1 i R /i , 2 i S /i)-(1,2-epoxypropyl)phosphonate monohydrate
